# The National Health Cluster in Yemen: assessing the coordination of health response during humanitarian crises

**DOI:** 10.1186/s41018-022-00117-y

**Published:** 2022-03-24

**Authors:** Sameh Al-Awlaqi, Fekri Dureab, Marzena Tambor

**Affiliations:** 1 Institute of Public Health, Jagiellonian University Medical College, Krakow, Poland; 2 School of Health and Related Research, The University of Sheffield, Sheffield, UK; 3 Institute of Research for International Assistance, Akkon Hochschule, Berlin, Germany; 4 Heidelberg Institute of Global Health, Heidelberg University, Heidelberg, Germany; 5 Health Economics and Social Security Department, Institute of Public Health, Jagiellonian University Medical College, ul. Skawińska 8, 31-066 Kraków, Poland

**Keywords:** Health Cluster, Yemen, Coordination, Conflict, Humanitarian health response, Evaluation, Health systems

## Abstract

Yemen has been facing political, economic and social challenges since 1990. The fragility of Yemen’s situation has led to a widespread conflict in 2015, resulting in the world’s largest humanitarian crisis. Amid the humanitarian catastrophe and the collapsing health system, a platform for coordinating humanitarian health response, called the National Health Cluster, has expanded its operations across the country. The study aims to evaluate the performance of the National Health Cluster in Yemen between 2015 and 2019. A qualitative research design was employed, and ten semi-structured interviews with key Health Cluster stakeholders were conducted. The study applied the Active Learning Network for Accountability and Performance in Humanitarian Action (ALNAP) guide to evaluating humanitarian action using the Development Assistance Committee (DAC) criteria. Six evaluation criteria were selected: relevance, effectiveness, efficiency, effects, connectedness and participation. Inputs from interviews were manually transcribed and then analysed using NVivo 12 software. The study results indicate that the Health Cluster in Yemen has contributed to saving lives and strengthening the local health capacities in diseases surveillance. In addition, its positive effect was evident in improving the humanitarian health response coordination.

Nevertheless, engaging health stakeholders, especially national organisations, was suboptimal. Exit strategies were lacking, while services to address mental health, non-communicable diseases, senior citizens and people with disabilities were not prioritised in the Health Cluster strategic plans and partners’ response. To ameliorate Health Cluster performance, revising its objectives and establishing a cluster-specific rapid response funding mechanism are pivotal. Furthermore, preparing the national health system for recovery and actively engaging all stakeholders in the Health Cluster’ response and strategic decisions would maximise its positive impact on Yemen’s health system and population.

## Background

The Republic of Yemen was established on 22 May 1990 following the merger between the Yemen Arab Republic (North Yemen) and the People’s Democratic Republic of Yemen (South Yemen) (UNDP 2013; ACAPS 2016). The Houthi rebels seized the capital Sana’a in late 2014 and then marched towards the south to apprehend President Hadi, who fled to the south and declared Aden the interim capital of Yemen in March 2015. The internal conflict took an international perspective when the Yemeni president escaped Aden to Saudi Arabia and requested military assistance from neighbouring countries and the United Nations (UN) Security Council to restore the legitimacy of his government. The 6-year conflict has deteriorated Yemen’s overall health and development outlook (Action Against Hunger 2019; Moyer et al. 2019), turning the country into the largest humanitarian crisis in the world (OCHA 2019b).

Fragmentation of the health system has emerged as a natural consequence of the protracted crisis since 2015. Two completely independent ministries of health manifested this fragmentation: one in the northern territories controlled by the de facto authorities, i.e. the Houthis’ government, and another in the southern part of the country run by the legitimate, internationally recognised government (Michael 2019; Pulitzer Center 2019).

The war had a calamitous impact on the health system, with more than half of the health facilities either not operational or destructed (WHO 2017b; Yemen Health Cluster 2017). The Ministry of Public Health and Population (MoPHP) has failed to disburse health workers’ salaries for several years (MSF 2017; Yemen Health Cluster 2017; Al-Awlaqi 2019). Epidemics such as cholera (declared the largest in the twentieth century) and diphtheria, as well as deteriorating levels of malnutrition and, most recently, COVID-19, burdened the collapsing health system (Dureab et al. 2020).

Before the escalation of the conflict in 2015, millions of Yemenis suffered from adverse social and economic situations; many were living below the poverty line, i.e. on less than $1.25 per day for basic needs such as food, shelter, or clothing (World Bank 2015; European Union 2016). As of 2021, around two-thirds of Yemenis desperately need humanitarian aid (OCHA 2021b). Furthermore, the latest Global Hunger Index Report 2020 states that Yemen is one of eight countries with an “alarming level of hunger”, including various forms of life-threatening malnutrition among children (Grebmer et al. 2020).

### The Cluster Approach

The concept of the Cluster Approach was first introduced in 2005 by the Emergency Relief Coordinator (ERC) of United Nations (UN) Office for the Coordination of Humanitarian Affairs (OCHA), Jan Egeland, to address the gaps in coordination and effectiveness in humanitarian response (Stoddard et al. 2007). The Cluster Approach was one of the pillars of the so-called humanitarian reform agenda, which came into effect following the inadequate and poorly coordinated humanitarian response to the crisis in Darfur and the Indian Ocean earthquake (OCHA 2007; International Council of Voluntary Agencies (ICVA) 2013). There are eleven clusters specialised in different humanitarian disciplines, i.e. preventing emergencies and mitigating their risks, preparedness and response to disasters and working in recovery and reconstruction phases (Inter Agency Standing Committee (IASC) 2015). The clusters’ titles and their lead agencies are listed in Table 1.

**Table 1:** Humanitarian clusters and their lead agencies, adapted from the IASC Cluster Coordination reference module (Inter Agency Standing Committee (IASC) 2015)

No.	Title of Cluster	Cluster Lead Agency
1	Emergency telecommunication	World Food Programme (WFP)
2	Education	United Nations Children’s Fund (UNICEF) and Save the Children
3	Early recovery	United Nations Development Programme (UNDP)
4	Camp coordination and camp management	International Organization for Migration (IOM)/United Nations High Commissioner for Refugees (UNHCR)
5	Water, sanitation, and hygiene	UNICEF
6	Shelter	International Federation of Red Cross and Red Crescent Societies (IFRC)/(UNHCR)
7	Protection	UNHCR
8	Nutrition	UNICEF
9	Logistics	WFP
10	Health	World Health Organization (WHO)
11	Food security	WFP and Food and Agriculture Organization (FAO)

Health Clusters exist in twenty-nine countries and territories with emergencies or protracted humanitarian crises and two regional coordination mechanisms in Syria and the Pacific (Global Health Cluster 2021). Its mandate is to provide a coordinated response and avoid gaps and duplication of humanitarian response during emergencies (WHO 2015).

### The Health Cluster in Yemen

In response to the failure of the national system to meet the increasing population health needs amid the escalation of conflict, many of the health systems’ functions in both sides of Yemen (north and south) were heavily supported by the UN agencies, international non-governmental organisations (INGOs), and national non-governmental organisations (NNGOs) (OCHA 2019b). These organisations coordinate their humanitarian health response via the Health Cluster, led by WHO, the Cluster Lead Agency (CLA) (Yemen Health Cluster 2018).

The Cluster Approach was first introduced in Yemen in 2009 following the resurgence of hostilities in the northern part of the country (Sa’adah wars), with the Nutrition Cluster being the first cluster to be activated (OCHA 2019a). In March 2010, the arrangement for establishing the Health Cluster was formalised and then activated in 2011 (OCHA 2010; Global Health Cluster 2018)

Since the beginning of the recent hostilities in 2015, the Health Cluster expanded its operations in what is known as “subnational hubs” or “subclusters” in the following areas: Sana’a, Aden, Ibb, Hodeida and Sa’dah (until early 2019). These hubs cover the twenty-two governorates of Yemen (OCHA 2021a).

The Health Cluster works with the MoPHP to deliver coordinated health response to the affected populations throughout the country. It has the following objectives (Yemen Health Cluster 2018):

1. To increase access of vulnerable populations, including internally displaced persons (IDPs), to MHSP covering life-saving and basic quality health services, through support to health system and community resilience
2. To strengthen preparedness, surveillance and response to communicable diseases outbreaks and epidemics, including immunisation for vaccine-preventable diseases
3. To deliver a principled and coordinated health response and promote an integrated approach with other sectors for a comprehensive humanitarian response with a focus on the most vulnerable districts
4. To improve access to reproductive, maternal, newborn, child and adolescent health services for vulnerable populations, including IDPs and the poorest and deprived segments in the society

When conducting this study in 2019, two prominent evaluations of the Cluster Approach at the global level were available (Stoddard et al. 2007; Steets et al. 2010). More recently, another two studies have attempted to determine the ideal level of coordination within the Cluster Approach in general and the role of the Health Cluster in the cholera response in Yemen, respectively (Clarke and Campbell 2018; Hopkins 2019). However, none of these studies evaluated the overall Health Cluster performance during the conflict period in Yemen.

## Methods and materials

This research aims to evaluate the national Health Cluster performance during the conflict period (2015–2019) in Yemen by employing a qualitative research design.

We conducted in-depth interviews with key informants who represented major health stakeholders in Yemen. To guide the interview and data analysis, we developed an analytical framework based on the Active Learning Network for Accountability and Performance in Humanitarian Action (ALNAP) guide on evaluating humanitarian action. ALNAP guide applies Organisation for Economic Co-operation and Development (OECD)’ Development Assistance Committee (DAC) criteria to fit the context of complex humanitarian emergencies. This evaluation guide combines best practices and provides a helpful instrument for evaluating humanitarian interventions for individual evaluators and organisations alike (ODI 2006).

To ensure that we cover the main methodological considerations in this research, we applied the Consolidated Criteria for Reporting Qualitative Research (COREQ)' checklist, which lists the most commonly used steps in qualitative research design (Tong et al. 2007a, b). The checklist aligns with the Enhancing the Quality and Transparency of Health Research (EQUATOR)' recommendations (EQUATOR Network 2015).

### Analytical framework and data collection instrument

We used six criteria (relevance, effectiveness, efficiency, effects, connectedness and participation). We excluded other criteria for the reasons detailed in Table 2.

**Table 2:** Description of ALNAP-DAC evaluation criteria used to design the study’s analytical framework (adapted from ALNAP guide 2006)

Criteria	Definition and comments
**Relevance**	It evaluates the synergies between the intervention, national priorities and local needs. It can be used in all evaluation types
**Effectiveness**	It indicates to what extent an intervention was successful in bringing about the intended results. It also implies timeliness and can be used in sectoral or organisational evaluations
**Efficiency**	It measures all resources (monetary and nonmonetary) required to achieve the desired output and compare alternatives to achieve the same output. Thus, it can be used in all evaluation types with adequate financial data
**Effects**	“Effects” is not a standard criterion, but it was intended to replace “impact”. “Impact” needs a comprehensive assessment that involves the affected population, which cannot be covered in this study. Alternatively, the study looked at various “effects” on the national health system, humanitarian response coordination, Cluster partners and a vulnerable population
**Connectedness**	It ensures that short-term interventions are linked with long-term plans and can be applied in evaluations that focus on coordination between organisations and stakeholders. It is adapted from the concept of “sustainability”
**Cross-cutting theme**	
**Participation**	Participation means that primary stakeholders (Cluster partners) must be fully engaged in all phases of developing and implementing health interventions Among the eight cross-cutting issues, participation was relevant to the case of Yemen as it reflects how active was the Cluster in engaging partners. In addition, it provides a glimpse of the coordination and synergies between the Health Cluster and its members and highlights the power dynamics within the Cluster
**Excluded ALNAP-DAC criteria**	
**Coverage**	Coverage was not selected because it needs identification of the people who benefited from humanitarian action and why they have benefited or excluded from that action (ODI 2006), which is beyond the scope of this study. However, as coverage has an indirect link with “effectiveness”, some aspects of the coverage, i.e. identifying vulnerable groups within the Health Cluster partners’ response, has been included in the study
**Coherence**	Coherence focuses on incorporating a humanitarian dimension into global policies. Measuring the coherence of the Health Cluster as a coordination mechanism against international policies is too broad and does not fit the purpose of this study
**Impact**	“Impact” was replaced by “effects”. Assessing impact needs a specific impact evaluation approach (Steets et al. 2009, 2010). It also requires collecting feedback from the affected population (Hallam 1998), which is beyond the researcher’s capacity. Previous evaluation reports of the Cluster Approach employed “effects” as a criterion instead of “impact” for comparable reasons (Steets et al. 2009)

The evaluation framework and criteria applied in this study were previously used in various humanitarian settings over the last two decades (ODI 2006). Specifically, they were applied in to evaluate the Cluster Approach at the global and national levels (Steets et al. 2010).

We used the analytical framework to prepare an interview guide for semi-structured interviews. The interview topic guide was divided into six themes corresponding to the six evaluation criteria; each criterion has specific, mostly open-ended, questions.

We developed the topic guide in English and piloted it before commencing actual interviews. A few revisions were added, mainly on the relevance (i.e. the cluster objectives were explicitly mentioned to give more clarity to the informants) and on the efficiency criteria, i.e. questions have been revised to reflect funding.

### Study population and sample selection

The population of this study covered various Health Cluster stakeholders, including the Ministry of Health, UN agencies, INGOs, NNGOs, and international health experts who work or have previously worked in Yemen between 2015 and 2019. These different stakeholder groups were selected to ensure a broad and diversified perspective for the study (Hallam 1998).

We followed a non-probability, purposive sampling, namely expert or stakeholder sampling, to identify key health stakeholders in Yemen. The selection of key informants was based on researchers’ previous national and international expertise in the Cluster Approach and humanitarian coordination. In evaluations and evidence-informed policy research, it is recommended to employ stakeholders sampling (Palys 2008).

Sixteen key informants fit the definition of the study population (Cluster coordinators, MoPHP officials, representatives of national and international NNGOs and international humanitarian experts). Out of the targeted sixteen informants, ten participated in the study (Table 3). Among those who did not take part, four did not respond to invitation e-mails.

**Table 3:** Characteristics of the recruited participants

Organisation	Gender
Health Cluster (former coordinator)	Male
NNGO	Male
NNGO	Female
Nutrition Cluster	Male
Health Cluster	Male
WHO	Male
OCHA	Male
INGO	Female
INGO	Male
International health consultant (former coordinator in Yemen)	Male

Despite the probability of introducing bias by the selected sampling technique, we attempted to include a diverse sample as possible among key health stakeholders to obtain a holistic picture about the Health Cluster in Yemen.

### Data collection

The principal investigator conducted the interviews between March and April 2019. Eight interviews with key informants residing in Yemen were conducted online. Two participants were interviewed face to face. Each interview lasted around 45 to 60 min. The interviews were audiotaped alongside manual notetaking. Audiotaped data were transcribed by manual typing into word documents (verbatim).

Eight interviews were conducted in the English language and two in the Arabic language, in line with the preferences of some participants. The latter was translated into English by the principal investigator before the analysis.

### Data analysis

The principal investigator uploaded all transcripts into NVivo software version 12 for Macintosh. At the beginning of the analysis, six themes (called nodes or codes on NVivo), which corresponded to the six evaluation criteria (relevance, effectiveness, efficiency, effects, connectedness and sustainability), were manually created under “codes>nodes” tab within NVivo software by the researcher.

An additional two themes (areas for improvement and challenges) were added to the list of themes that were not on the main list of themes as they were found relevant during the analysis.

The study findings were analysed and fact-checked by the following:

1. Reflexivity: the author’s own experience in Yemen as a public health expert — and a previous cluster co-coordinator — was taken into consideration.
2. The results were compared against the available body of literature (mostly grey literature) on coordination mechanisms in humanitarian settings.
3. The anonymised results were checked by public health specialists from Yemen and other countries with previous experiences in humanitarian health response in Yemen.

### Ethical considerations

The study obtained ethical approval from the School of Health and Related Research (ScHAAR) Ethics Committee before interviews. After receiving a detailed participant information sheet, participants provided their written consent to participate in this study and to use their anonymised responses in future publications.

## Results

### Relevance

Relevance is defined as compliance of the Health Cluster objectives with the national health priorities and the local health needs of the population (ODI 2006).

Many key informants agreed that the Health Cluster’ objectives in Yemen were, to certain extent, relevant to the national health priorities and the needs of the affected population. These objectives, nevertheless, did not translate into timely response due to weak decision-making power within the Health Cluster itself, especially at the level of its subnational hubs.

Another informant, an international health expert, indicated that these objectives were relevant yet limited in scope to life-saving interventions.

*“ …But I think these (the Cluster objectives) are more or less remaining in the field of life-saving, […] the needs are much more, much bigger than actually what all these actors together can deliver.”* **(Informant #7)**

The informants’ opinions about the objective of delivering a principled and coordinated health response and promoting an integrated approach were mixed. Informants from INGOs thought that there were bottlenecks in delivering a principled health response due to the political influence of the national authorities on the Health Cluster decision-making process, i.e. national authorities or parties to the conflict grant access to field areas only if the Health Cluster and its partners abided by the preconditions of these authorities. These preconditions, on some occasions, contradicted the humanitarian principles:

*“I think the Health Cluster is struggling […] in a politically polarised environment such as Yemen setting. (It was) so difficult to work in a fully principled way because sometimes a lot of humanitarian principals were compromised by the Health Cluster and partners in return to access”. ***(Informant#9)**

### Effectiveness

Effectiveness answers the question of whether the Health Cluster activities achieved their desired purpose at the right time. When questions about effectiveness were asked, four sub-themes emerged, i.e. the overall effectiveness, the timely response, the Ministry of Health fragmentation and the multisectoral programming.

The opinions of key informants on the overall effectiveness of the Health Cluster performance during the last 3 years were dissimilar. Cluster coordinators and respondents from INGOs, NNGOs and UN organisations believed that the performance was unsatisfactory and needed further improvement (first group). The second group, nonetheless, represented by other UN agencies and international experts, witnessed a compelling performance.

Moreover, among those who believed that the performance was not satisfactory, many informants have identified specific gaps in the Health Cluster work. They criticised the interference of the Cluster Lead Agency (CLA) in the Health Cluster work and the unsatisfactory level of Health Cluster’ transparency in sharing its work plan and budget. Furthermore, the Health Cluster’ overambitious targets and the political influence of national authorities on the Health Cluster’ work were other significant drawbacks that negatively impacted the effectiveness of the Health Cluster.

*“I felt that sometimes the coordinators […] were already taken by parties to the conflict […] they were not representing the voices of all partners”. ***(Informant#9)**

*“They (decision makers) were not present at Aden hub; they were in Sana’a. Remote management was difficult, and the response was difficult, slow or not up to the need”. ***(Information #10)**

Among those who witnessed satisfactory performance, prevention of deaths, proper coordination among the health stakeholders and clusters and provision of subsidised or free health services in the Health Cluster partners’ response were the areas the Health Cluster performance was evaluated as effective in addressing them:

*“I can say it with a very good feeling that we prevented massive deaths […] hundreds of thousands of deaths could happen if you have no health system and the country is actually in humanitarian crisis; this did not happen”. ***(Informant #7)**

*“I think the Health Cluster has done a good job […] we developed minimum quality standards matching the minimum service package adopted by MoPHP. Those minimum quality standards also go and in line with the SPHERE standards”*. **(Informant#5)**

Many informants commonly stated that the Health Cluster response was not timely. They attributed that to the division within the Ministry of Health and the lengthy bureaucratic procedures of the new and existing political parties to get health projects approved. This division resulted in two different procedures corresponding the two sides of the country. These procedures have to be followed and coordinated at two parallel levels by the Health Cluster’ partners, which was very challenging:

*“Now we have a national authority for managing or coordination of the humanitarian assistance they call it NAMCHA*^1^*[…], I am talking about de facto authorities. There are many layers; too many coordination and difficulties the health partners were facing”. ***(Informant#5)**

*“Two Yemeni governments, South and North, two governments are controlling different geographical areas and each claim they are controlling the whole of Yemen, which weakened the decision-making among partners when they tried to abide by policies of one or another entity, which affected the response”. ***(Informant #1)**

### Efficiency (funding)

According to ALNAP definition, efficiency assesses how project inputs that have monetary value were converted into results, taking into consideration whether the results were maximised for given inputs. It may entail comparison with an alternative to assess the most efficient approach to implementing an intervention (ODI 2006).

Due to the lack of adequate financial data, respondents could not comment on the specific budgets allocated for the Health Cluster nor were able to identify a more efficient alternative. Therefore, answers to this theme were focused on “funding” and whether it was sufficient, rather than “efficiency” in its economic interpretation. It also addresses the role of the Health Cluster in governing the health sector’ funds via the Yemen Humanitarian Response Plan (YHRP).

Most informants indicated that there were no direct funds allocated for the Health Cluster to provide health response:

*“There were no direct funds to Health Cluster, there were funds that went to the main stakeholders such as UNICEF, WHO or UNFPA, but the Cluster has no direct funds”. ***(Informant #1)**

Some informants from NNGO and UN organisations also indicated that the Health Cluster coordination budget was not matching the needs of the population. Other informants among the INGOs confirmed that the Health Cluster could not mobilise resources on some occasions, especially in areas with cholera resurgence:

*“The fund for the Health Cluster is still not enough, because it is not proportional to the existing gap or the programme”. ***(Informant #8)**

*“After we agree with the Ministry of Health of both sides and with the partners […] (and) have a clear framework and plan, indicators, and activities, then we have to cost these activities. Based on what we call it severity matrix […], (It has) health system indicators, the availability of service, human resource, the functionality of the health facilities, and so on”*. **(Informant #5)**

### Effects

Effects, derived from the term impact, consider all negative and positive encounters resultant from the Health Cluster performance and activities during the appraised period.

Most key informants agreed that there were positive effects on the health system because the Health Cluster partners’ response allowed the health system to provide Minimum Health Service Package (MHSP), strengthened the local capacity and advocated for adequate funding:

*“I think without the Health Cluster, the situation in Yemen would have been much worse for the national health system”. ***(Informant #7)**

*“If you compare between HeRAMS*^2^*2016 and 2018, I can say there was a good improvement in the health system inputs […]. That is how the Health Cluster helped in maintaining the functionality of the national health system”. ***(Informant #5)**

On the other hand, one of the adverse effects of Health Cluster on the health system, as emphasised by some key informants who represented international experts and NNGOs, was that it might have contributed to the shortage of senior MoPHP staff by recruiting them to work for the Cluster or various UN agencies. Others argued that the huge incentives will have a detrimental effect on health staff availability in the future:

*“We are also responsible for the tragedy of the national health system, why? […] ministries are increasingly getting weaker by the time, and we are contributing to this weakness. Most of our staff come from the Ministry, so we are actually depleting the Ministry of Health from its staff”. ***(Informant #7)**

*“I think this intervention (humanitarian agencies paying monetary incentives for health workers) was a bit risky, because if the health worker used to receive high incentives and those humanitarian partners stopped their incentives after the war (when the funds ceased), I am afraid that health service provision in these health facilities would collapse […] even if the government pays their salaries regularly”. ***(Informant#2)**

### Connectedness

Connectedness addresses the inclusion of exit strategies in the Health Cluster partners’ response during the last 3 years in Yemen. Exit strategies are plans to sustain the health intervention once the donor funding is over and the overall health situation is improved, i.e. as a result of peace and stability.

Responses from key informants regarding connectedness were diverse. These responses can be categorised into three categories: (a) no exit strategy incorporated, (b) exit strategies were included to some extent with limitations and (c) a paradoxical effect on connectedness.

Regarding the first category, a few informants from NNGOs and Health Cluster indicated that there were no exit strategies in the Health Cluster partners’ projects, and that during emergencies — which is the case in Yemen — the transition to development is not a priority

*“When we plan for humanitarian response, we do not think about sustainability because it is not a recovery intervention, it is just to provide first-line interventions to cover the acute needs, so talking about sustainability is difficult and not realistic”*. **(Informant#2)**

In the second category, i.e. exit strategies were included with limitations and challenges, the informants (INGOs, international experts and the Clusters) mentioned examples from the Health Cluster partners' experiences to support this statement. For instance, the ongoing capacity building activities of MoPHP staff at the facility and community levels during the conflict:

*“Whoever stays in the country, the human resource, they are now receiving incentives […] training, […] there is a capacity building. These things can be sustained even if the Health Cluster partners withdraw”. ***(Informant #5)**

The third category of informants from INGO, NNGOs, and international experts indicated that the Health Cluster partners’ response had, nonetheless, a paradoxical effect which adversely affected the connectedness aspect. Operating mobile health clinics outside health facilities for a longer time and paying high salaries or incentives to NNGOs and MoPHP staff were among the examples mentioned:

*“The government, yes, they were in favour of using mobile clinics in many locations for whatever reason, but no, […], this [ mobile clinics’ approach] is not going to take the Health Cluster interventions to sustainable solutions”. ***(Informant# 4)**

### Participation

During interviews, the key informants were asked about their views regarding motivation and the level of engagement of health stakeholders in the Health Cluster activities such as meetings, joint response plans and prioritisation of affected areas.

Many informants, except those who represented UN organisations and the Health Cluster, confirmed that the level of participation and representation of health stakeholders in the Health Cluster was very minimal, especially the national NGOs and local health authorities. Informants emphasised that these NGOs were not given an equal chance to participate in the decision-making process of the Health Cluster.

*“I used to attend civil society organisations’ meetings, and I was surprised that many organisations did not even know about the Cluster system”*. **(Informant #3)**

Furthermore, some informants from national NGOs believed in a vicious cycle of underrepresentation, underfunding and poor capacity. These issues hindered the active participation of national NGOs in the Health Cluster:

*“...I found the national NGOs’ role was not strong or not effective in the Cluster like (compared to) international NGOs, maybe because of the low capacities […], the lack of confidence within their capacities or they thought their role was not important, as most of the discussions became dominant by international NGOs”. ***(Informant #2)**

**Fig. 1:**
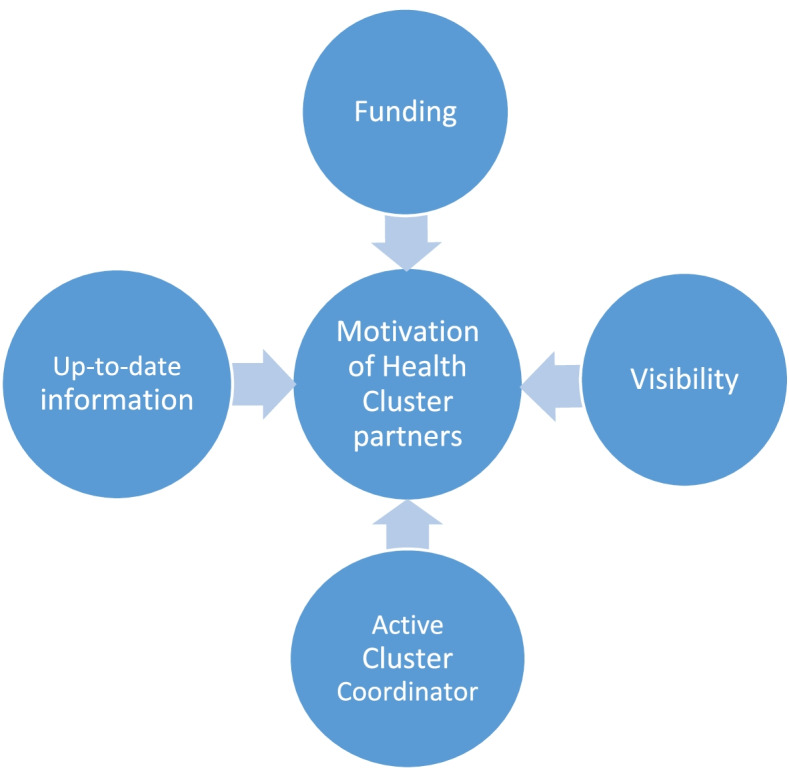
The main motivations of health stakeholders to actively participate in the Health Cluster meetings in Yemen

All informants agreed that there were four main reasons (Fig. 1) which motivated the partners to be active Health Cluster members, namely staying well-informed with the field situation, getting visible in national and international humanitarian platforms, and, most importantly, having priority access to humanitarian aid funds.

### Challenges

This theme emerged during the interviews. Two main categories of challenges were discussed during the interviews; these were the following:

A- **Governance and capacity:** fragmentation within MoPHP and the weak capacity of its staff, especially in management and planning capacities, were among the main challenges indicated by many respondents. Furthermore, the strong centralisation of the Health Cluster was a challenge, too, as it prevented timely decision-making within its peripheral subnational hubs. Finally, the limited financial resources of the Health Cluster hindered deploying a swift response to those who need it:
  *“The problem is the centralisation. Which means that there are no decisionmakers at the sub-cluster level. The decisions come from the Cluster itself in Sana’a”. ***(Informant #10)**

Moreover, informants, mainly INGOs and NNGOs, had to undergo parallel coordination and authorisation procedures at the two sides of the country, which was a time-consuming process that hindered their timely response. For instance, the newly emerged coordination structure within the de facto territories (NAMCHA) has delayed health projects’ implementation because of the very lengthy bureaucratic procedures it applies to get projects approved.

*“So, if you have a project for one year, can you imagine losing four months in coordination? Then […] when you go to the governorate you have to repeat the same process with NAMCHA branch, with the national security, with the governor office, with the governorate health office, with the (district) health office and so on. I think this is one of the most important things that we need to sort out to help the Health Cluster performing well”*. **(Informant #5)**

B- **Access and coverage of health services:**

The informants from INGOs, NNGOs and Health Cluster expressed that they had difficulty in accessing many deprived areas for various reasons, e.g. security issues, checkpoints and local authorities, which was one of their biggest challenges as it hindered the implementation of health response.

Some informants mentioned the unaffordability of health service to many vulnerable populations, because of user fees and cost of treatment, as another tough challenge that hindered vulnerable people from receiving the treatment they need:

*“We note that in the North and the South- the de facto power [ in the North] and the legitimate government in the South-, are putting conditions on the NGOs to access populations”*. **(Informant #9)**

### Areas for improvement

According to the key informants, specific areas within the Health Cluster performance can be improved. These areas can be organised under four headings: (1) strengthening leadership, decentralisation, and effectiveness, (2) improving participation and inter-Cluster collaboration, (3) transition to development and lastly, (4) cross-cutting recommendations.

1. **Leadership:** First, more delegation and decision-making power should be given to the subnational hubs, including financial independence. Second, to deliver a principled response abided by humanitarian principles, the Health Cluster should maintain its full autonomy and impartiality without interference from CLA or local authorities. Third, strengthening advocacy for unrestricted access to affected areas is paramount.

Some informants stated that it is also crucial to clarify the role of the Health Cluster to the local authorities and stakeholders. The informants expressed their need to have a strong, neutral and impartial cluster coordinator who can act with power and integrity, despite the enormous political pressure, to represent the best interests of the Health Cluster partners among the national authorities and international actors.

*“We want strong (Health Cluster) leadership; strong leadership means we need fighters in this position (Cluster Coordinator) to fight on behalf of the group (Health Cluster partners)”*. **(Informant #9)**

2. **Participation:** Participation and inter-Cluster collaboration can be improved, according to informants from the Health Cluster and INGOs, by strengthening inter-Cluster coordination mechanisms, especially with the Nutrition Cluster. Informants, mainly INGOs, emphasised the importance of representing NGOs in the Health Cluster technical working groups and the Strategic Advisory Group (SAG). Moreover, in the opinion of some participants, the Health Cluster should encourage and motivate the MoPHP to play a more active role in the Health Cluster plans and response.
3. **Transition to development:** which is required to sustain the current health services and prepare the health system for the next phase, i.e. health system recovery. Participants, mainly from Health Cluster and international experts, urged that it is inevitable to invest in the public health sector and national NGOs to build their capacities and to improve the cost-effectiveness of health interventions, especially in areas with no current conflict. Some informants stated that there is a need to find an approach to retain senior and qualified ministerial staff in their positions within MoPHP.
4. **Cross-cutting recommendations:** One informant supported mainstreaming gender equality in the Health Cluster by recommending a female staff for the position of Cluster Coordinator. This informant stated that considering gender issues within the Health Cluster management will ensure that maternal mortality, gender-based violence (GBV) and local cultural sensitivities that affect health are taken care of effectively.
  *“We should consider that the Cluster Coordinator (is a female), at least. We should have more females because a big part of life-saving humanitarian interventions has to do with women”. ***(Informant #7)**

## Discussion

This study provides insights into the Health Cluster performance in Yemen between 2015 and 2019, intending to use these reflections to improve the coordination of the humanitarian health response in Yemen.

Previous studies attempted to assess the ideal level of coordination within the clusters in general (Clarke and Campbell 2015) and the role of the Health Cluster, among other coordination platforms, in the cholera response in Yemen (Hopkins 2019). However, there were no specific evaluation studies on Yemen’s Health Cluster performance during the conflict period. Therefore, this study fills a unique niche within the literature concerning health coordination in fragile and conflict-affected settings with its primary focus on Yemen. In their study on the Cluster Approach, Clarke and Campbell, which included Yemen among seven other countries (Clarke and Campbell 2018), found that the participation of NNGOs in the Cluster was extremely weak, which represented one of the main drawbacks of the Cluster system in these countries.

The results of this study indicate that the Health Cluster in Yemen was (and still is) a vital coordination mechanism for the humanitarian health response during the appraised period (2015–2019), effective in achieving most of its objectives and reaching many people in need.

First, the Health Cluster objectives were partially relevant to the context of Yemen between 2015 and 2019 and, to some extent, were in line with the national health priorities and the urgent health needs of the population. Nevertheless, these objectives were limited to life-saving interventions, and they lacked an explicit “transition to development” perspective, as many informants in the study indicated.

Second, the results of this study show that the Health Cluster was partially effective in achieving its objectives and its intended purpose during the assessed period.

The views were diverse; those who run and participate in the Health Cluster had a more critical view, perhaps because of their extensive involvement in the Health Cluster at the micro level. While those from UN organisations, in their views, may have reflected on the overall achievement of the Health Cluster from the macro level, i.e. life-saving interventions, the number of people reached and other overall targets.

Even though the Health Cluster's interventions successfully prevented potential deaths and treated morbidities, timely health response was a challenge due to many factors. The ongoing conflict, limited financial resources allocated for the Health Cluster and Yemen's North-South divide, to name a few.

Third, based on the results of this study, it can be concluded that the Health Cluster performance would have benefited from robust decentralised structures at the subnational hubs (Hopkins 2019). According to the informants, although these subnational hubs were established during the conflict in 2017 (Yemen Health Cluster 2017), they lack decision-making power. Moreover, the Health Cluster did not sufficiently consider the North-South divide, i.e. two ministries of health, which might have necessitated a different approach. For example, establishing two national clusters with full autonomy ensures harmonised, equitable and timely health responses to those who need it in both areas.

Fourth, the Health Cluster capacity to deploy field assistance during an outbreak or an acute health emergency was severely restricted by the limited financial resources. Many key informants indicated that it is vital that the Health Cluster has its budget, independent of donors’ proposal windows, to respond to emergencies at their early stages. Then, additional projects with larger budgets can back the initial response once the donors are on board and all bureaucratic procedures to deploy more significant funds are finalised.

Moreover, the study showed that the Health Cluster performance between 2015 and 2019 had many positive and negative effects at various levels of the national yet divided health system, humanitarian response coordination, health stakeholders and the population in need. The Health Cluster partners’ response provided tremendous support to the health system and ensured the continuity of life-saving health services (Yemen Health Cluster 2017). In addition, financial incentives for health workers, which were included in many of the partners’ health programmes, were a significant step toward maintaining a minimum level of health system functionality.

The role of the Health Cluster in supporting health management information system, i.e. strengthening the early warning system, has improved the surveillance capacity within the MoPHP. The same finding was confirmed in another study about MoPHP preparedness to outbreaks in Yemen (Dureab and Jahn 2019).

Furthermore, the Health Cluster’s effects on the humanitarian coordination were described as positive and considered by many informants. However, the humanitarian organisations, including the UN agencies and the Health Cluster itself, may have contributed to the shortage of competent senior staff at the ministerial level by recruiting them directly to work for the UN organisations. Moreover, some informants added that UN recruitment offers a more competitive remuneration package compared to the public sector.

The Health Cluster provided a platform for effective health coordination, attempted to build partners’ technical capacities and prioritised them for funding opportunities. Nevertheless, power dynamics within the Health Cluster played a negative role on the national partners as NNGOs felt neglected and not involved in the strategic decision-making process compared to their international counterparts.

Active engagement of national NGOs feeds into the broader strategy of the Global Health Cluster, which stresses the importance of adopting a “localisation” approach to strengthening the national capacities, i.e. investing in national organisations to expand and sustain health services in the long-term (WHO 2017a). However, there were limitations in addressing gender within the Health Cluster, with a persistent trend of limited involvement of females at the senior leadership, i.e. Cluster Coordinator’ position in the capital was occupied exclusively by males.

Finally, the results of this study indicate that the focus of the Health Cluster on life-saving interventions and not prioritising plans for exit strategies or health system recovery might be an area that needs improvement. Establishing a more vital link between the current health interventions and the longer-term plans for health system recovery and transition to development, the so-called humanitarian-development nexus, is crucial and should be prioritised (Qirbi and Ismail 2017), especially in stable areas where there is no actual conflict, i.e. southern and eastern governorates. This approach, of ensuring connectedness, is in line with the Global Health Cluster, WHO and IASC recommendations (WHO 2016, 2017a; IASC 2019). This fits within the New Way of Working, a partnership approach specifically designed to strengthen the humanitarian-development-peace nexus by building collaborative partnerships among UN organisations, civil society organisations and governments to address the root cause of conflict and fragility and their consequences on the communities (OCHA 2017).

### Recommendations for policy and research

The study recommends several actions to be taken from policy and research perspectives. It is crucial to understand that the implementation of these recommendations could be challenged by the political and security situation and by the global partners’ conflicting agenda for Yemen. However, these recommendations can act as an umbrella to streamline efforts to improve the Health Cluster performance in Yemen.

It is pivotal to refine the Health Cluster objectives and further align them with the national priorities and population emergent needs from a policy perspective. The key recommendations are establishing a rapid funding mechanism managed by the Health Cluster, strengthening the local capacities and supporting the health system in transition to recovery and development.

Concerning the recommendations for research, the Health Cluster will benefit from further studies which involve measuring the effectiveness and cost-effectiveness of the health sector interventions coordinated by the Health Cluster. Impact evaluation would provide accurate figures on the medium-to-long-term impact of the Health Cluster, especially on the vulnerable communities and the national health system. Bringing lessons learnt and success stories from other countries with similar contexts, especially those related to health system recovery and humanitarian-development nexus, might improve the Health Cluster strategic directions and align its objectives to population needs. The recommendations for policy and research are summarised in Table 4.

**Table 4:** Recommendations for policy and research

Study outcome	Recommendations		Organisation
	Policy	Research	
**Relevance:** The Health Cluster objectives, although they were relevant (to some extent) to the context in Yemen, they had short-term and emergency perspective. Some vulnerable groups and illnesses need specific activities to address them.	- Cluster objectives can undergo a review process by all Cluster members to review their relevance and update them according to the gaps and needs. - Include specific objectives to emphasise health system strengthening and recovery outcomes. - Inclusion of objectives (or sub-objectives) to support people with special needs and mental health interventions.	- Further research on the best approach to identify relevant objectives and to measure their effectiveness and efficiency is needed.	Health Cluster, WHO as a CLA
**Effectiveness:** The Health Cluster was effective in delivering its activities according to objectives, but not on a timely basis. Neutrality and independence of the Health Cluster were not protected on some occasions.	- There should be a mechanism to provide a rapid response within the authority and budget of the Health Cluster to provide a swift response to emergencies. - Equal representation and delegation of decision-making power to leaders of subnational hubs. - Strengthening the Health Cluster leadership and financial independence. - Clarifying the role of the Health Cluster in all operational areas.	- Cost-effectiveness analysis of the health sector response coordinated by the Health Cluster would provide evidence to invest in the most effective interventions.	Health Cluster, WHO as CLA, OCHA, IASC
**Efficiency (funding):** The Cluster funds were enough to cover its basic coordination tasks, but not to respond to emerging outbreaks or displacement needs.	- The Health Cluster would benefit from having an independent financial mechanism to rapidly respond to emergencies at early stages.		Health Cluster, WHO, OCHA, donors
**Effects:** The Health Cluster gave suboptimal attention to MoPHP senior staff’ capacity, persons with disabilities, senior people, mental health, and chronic disease.	- Finding an approach to “second” MoPHP senior staff while maintaining their salaries and benefits. - Establishing and expanding activities to tackle chronic diseases, geriatric, and mental health and to prioritise enough assistance to people with disabilities.	- Impact evaluation research, which could include the perspective of the affected population within its design.	IASC, Health Cluster, WHO, UNICEF and OCHA, MoPHP
**Connectedness:** - The inclusion of exit strategies to sustain health services after the end of donors’ support was not included in the Health Cluster’ partners responses.	- The Health Cluster should encourage donors to include specific sections on exit strategies in health project proposals. - The Health Cluster shall work with MoPHP and all health stakeholders to draft and implement a health system recovery plan.	- Research to evaluate projects and to document successful stories (good practices) in sustaining the health services after concluding aid funds.	Health Cluster, SAG, UNFPA, OCHA, donors
**Participation:** - Participation and equitable representation of all partners in the Health Cluster’ decision-making process was unsatisfactory.	- The composition of SAG should be reviewed to ensure fair representation of all partners. - The process of decision-making within the Cluster should be clarified, standardised, agreed upon, and circulated to all partners upon their final collective consensus.	- Regular partner surveys, with action points to follow the outcomes. - Research on the role of observers (e.g. MSF) and the private sector in the Health Cluster.	Health Cluster, WHO, OCHA, MoPHP

### Limitations of the study

- The Health Cluster subnational hubs were not adequately represented in this study. Key informants came from the leading national hubs in Aden and Sana’a. There was difficulty in accessing subnational coordinators due to communication issues.
- The final sample size (10 interviews out of 16) might be small to draw representative conclusions. Nevertheless, poor phone and Internet connectivity, unstable security status and North-South divide (and the sensitivity around it) contributed to not reaching sixteen participants. Moreover, making “generalisation” or “statistical inference” was not the primary aim of this study, nor making objectivity judgments about the sample.
- The researcher could not organise the interviews with the current MoPHP officials. However, two key informants were former senior MoPHP staff at the ministerial level.
- The criterion of “efficiency” was not investigated in depth due to the lack of specific financial information regarding the funds allocated for coordination tasks of the Health Cluster work. However, the study attempted to answer basic questions about funding in terms of analysing the performance of the Health Cluster against the available known budgets (e.g. YHRP).
- Inconsistencies in using a single language in administering interviews might affect the quality of the transcripts. Thus, some critical concepts might be lost during translation, i.e. translating the two Arabic interviews to English.
- From an accountability perspective, the people in need are the end user of the Health Cluster partners’ field interventions, and their opinions could have benefited the findings of this evaluation study (Hallam 1998). However, the limited study scope combined with the difficulty in accessing the field in Yemen prevented the incorporation of interviews with the affected population within the study design.
- The role of Health Cluster observers - Médecins Sans Frontières (MSF), ICRC, and the private sector in the humanitarian coordination was not addressed because the study focused on full cluster members.
- Females might be underrepresented in the study. One contributing factor is that most cluster leads are predominantly males (at the study time). Females who participated in this study represented one NNGO and one INNGO.
- The principal investigator’s previous experience in the Cluster Approach in Yemen might have created bias during data collection and analysis. Nevertheless, comparison with other studies on humanitarian health coordination and examination of results by external health experts were applied to minimise the researcher’s bias.
- Limited scholarly work, especially peer-reviewed, on the Health Cluster in Yemen

## Conclusions

In this qualitative study, we attempted to retrospectively evaluate the Health Cluster performance in Yemen, covering 2015–2019. The study fills an important gap in the scholarly, peer-reviewed work related to the Health Cluster in Yemen. Overall, this study stresses the importance of effective health coordination during emergencies to save lives and strengthen local health systems. The response coordinated by the Health Cluster was adequate, with room for improvement. Despite the ongoing conflict, constrained access and division within the government, the Health Cluster’s response has succeeded to reach many populations in need and was in line, to a considerable extent, with the essential, life-saving health priorities. Moreover, despite the tremendous constraints faced, the Health Cluster has effectively coordinated a joint response with other clusters (Nutrition, WASH and FSL). For example, the cholera outbreak response and the integrated famine risk reduction response. Finally, the Health Cluster has worked closely with both governments to maintain the minimum health service package. However, the package was not comprehensive enough to fully include mental health services or respond to specific population groups’ needs, such as disabilities, chronic diseases or senior citizens.

## Data Availability

Not applicable.

## Footnotes

1 NAMCHA: National Authority for the Management and Coordination of Humanitarian Affairs

2 HeRAMS: Health Resources Availability Monitoring System
